# Disarming the Hsp70–Bim Alliance: Small‐Molecule and Peptidic Disruptors of a Chaperone‐Apoptotic Switch in Cancer

**DOI:** 10.1002/open.202500479

**Published:** 2025-12-12

**Authors:** Emadeldin M. Kamel, Mohammed Al‐zharani, Lina M. Alneghery, Noha A. Ahmed, Faris F. Aba Alkhayl, Al Mokhtar Lamsabhi

**Affiliations:** ^1^ Chemistry Department Faculty of Science Beni‐Suef University Beni‐Suef Egypt; ^2^ Department of Biology College of Science Imam Mohammad Ibn Saud Islamic University (IMSIU) Riyadh Saudi Arabia; ^3^ Physiology Division Zoology Department Faculty of Science Beni‐Suef University Beni‐Suef Egypt; ^4^ Department of Medical Laboratories College of Applied Medical Sciences Qassim University Buraydah Saudi Arabia; ^5^ Departamento de Química and Institute for advanced research in chemical Science (IAdChem) Facultad de Ciencias, Módulo 13 Universidad Autónoma de Madrid Madrid Spain

**Keywords:** allosteric inhibitor, bim, cancer resistance, chaperone addiction, hsp70, protein–protein interaction

## Abstract

Heat‐shock protein 70 (Hsp70) is a ubiquitous stress chaperone whose over‐expression confers treatment resistance in many cancers. Recent structural and mechanistic work has uncovered an unexpected survival circuit: the proapoptotic BH3‐only protein Bim binds a nucleotide‐sensitive groove on the Hsp70 nucleotide‐binding domain, sequestering itself from Bax/Bak while allosterically accelerating Hsp70's ATPase cycle and stabilizing oncogenic clients. This Hsp70–Bim protein–protein interaction (PPI) is enriched in tyrosine‐kinase‐inhibitor (TKI)‐resistant chronic myeloid leukemia, endocrine‐refractory breast cancer, glioblastoma, and other “chaperone‐addicted” tumors, making it a selective vulnerability rather than a housekeeping liability. Early linear and stapled BH3 peptides proved the groove is drug‐addressable but suffered from poor pharmacokinetics. A fragment‐assisted screen then delivered a phenalene‐dicarbonitrile chemotype, S1g‐2, and optimized analogs that displace Bim with sub‐micromolar potency, dismantle Hsp70–client hubs, and resensitize resistant xenografts to imatinib or tamoxifen without global proteostasis collapse. Orally bioavailable wedges thus convert a seemingly flat chaperone surface into an actionable checkpoint. This review integrates structural biology, assay technology, and medicinal chemistry to chart the rise of Hsp70–Bim inhibitors, evaluates combination strategies with BH3 mimetics, TKIs, and proteasome inhibitors, and highlights remaining challenges—cross‐isoform breadth, species‐relevant toxicology, biomarker‐guided dosing, and potential impacts on antiviral immunity. Future directions include covalent or macrocyclic wedges, degrader hybrids, and adaptive pulse‐dose regimens guided by proximity‐ligation assays. Collectively, chemical disarming of the Hsp70–Bim alliance exemplifies how precision targeting of chaperone PPIs can recalibrate apoptotic thresholds and unlock new therapeutic space in oncology.

## Graphical abstract

Targeting a nucleotide‐sensitive groove on Hsp70 that binds the Bim BH3 helix, we integrate structures, biophysics, and SAR from peptides, fragments, and phenalene‐dicarbonitrile “wedges.” These disrupt the Hsp70–Bim complex with sub‐µM cellular engagement and in vivo activity while sparing Hsp90/mortalin. We outline biomarkers and combination strategies for “precision proteostasis” therapeutics.

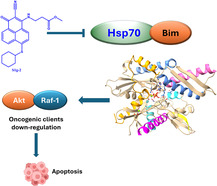

## 1 Introduction

**FIGURE 1:**
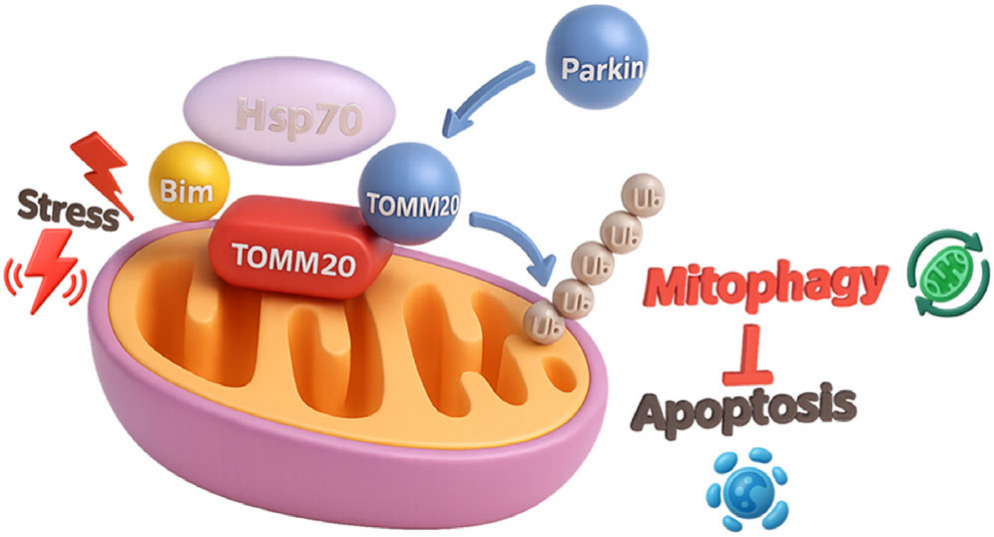
Hsp70–Bim–TOMM20 axis diverts Bim from apoptosis toward Parkin‐mediated mitophagy [3]. Under cellular stress, cytosolic Hsp70 binds the BH3‐only protein Bim and shuttles it to the mitochondrial outer membrane, where the complex docks on the import receptor TOMM20. The Hsp70–Bim platform recruits the E3 ubiquitin ligase Parkin (blue), which ubiquitinates TOMM20. Ubiquitylated TOMM20 triggers PINK1/Parkin‐dependent mitophagy, promoting selective removal of damaged mitochondria (green recycling symbol), and thereby suppressing Bax/Bak‐driven apoptosis. This noncanonical pathway explains how tumor cells can tolerate high Bim levels while escaping mitochondrial outer‐membrane permeabilization, adapted with premission [3]. Chemical‐shift perturbations map a hydrophobic triad in Bim (Leu94, Ile97, Phe101) that inserts into a pocket formed by Hsp70 Phe92 and Leu139, while the acidic side chains Glu90 and Asp98 form bidentate hydrogen bonds with the chaperone's Lys71 and Lys732023, Springer.

Heat‐shock protein 70 (Hsp70; HSPA1A/B) is the archetypal ATP‐dependent chaperone that safeguards the proteome during stress, yet it is also markedly over‐expressed in many tumors, where its buffering capacity thwarts apoptosis and fuels oncogenic signaling [1, 2]. Under acute stress, Hsp70 escorts the BH3‐only protein Bim (BCL2L11) to the mitochondrial outer membrane, where the complex docks on the import receptor TOMM20. This noncanonical partnership forms a landing pad for the E3 ligase Parkin, leading to TOMM20 ubiquitination and activation of PINK1/Parkin‐dependent mitophagy (Figure 1) [3]. In doing so, Hsp70 effectively diverts Bim away from its classical role—activating Bax/Bak and neutralizing prosurvival Bcl‐2 homologs—to a quality‐control pathway that removes damaged mitochondria. Such rewiring helps explain why cancer cells can tolerate high levels of Bim without triggering Bax/Bak‐dependent mitochondrial outer‐membrane permeabilization: Bim is sequestered into mitophagy rather than apoptosis [4].

In 2020, Guo et al. demonstrated that Bim's BH3 domain binds a previously unrecognized groove on the nucleotide‐binding domain (NBD) of Hsp70, converting the chaperone itself into a BH3 receptor [4]. NMR mapping located the interface opposite the ATP pocket, and biochemical assays showed sub‐micromolar affinity that preferentially targets the ADP‐bound, client‐embracing conformation of Hsp70 [4, 5]. Functionally, Bim acts as a positive cochaperone, accelerating Hsp70 ATPase activity and stabilizing oncogenic clients such as AKT and Raf‐1, even while being sequestered from its proapoptotic partners [4, 6]. This duality transforms Bim from a pure executioner into a shield for cancer cells.

**FIGURE 2:**
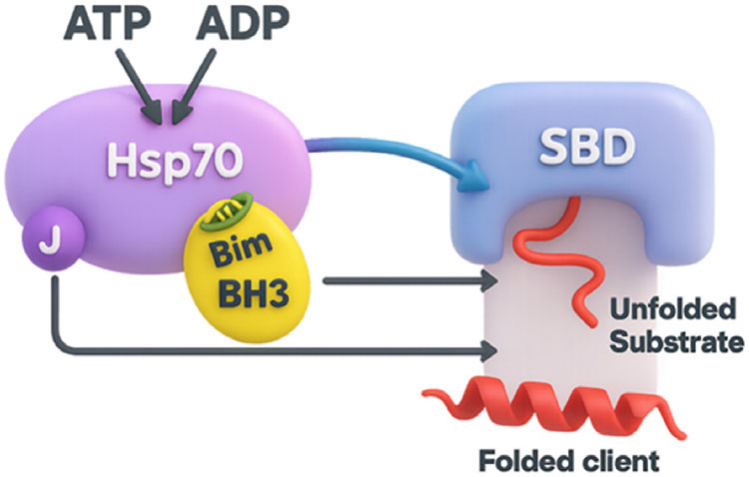
Bim licenses the Hsp70 chaperone cycle to fold and stabilize oncoprotein clients. A stylized 3D cartoon of Hsp70 depicts its nucleotide‐binding domain (NBD) accepting ATP and releasing ADP, while the substrate‐binding domain (SBD) captures an unfolded substrate (red ribbon) and releases it in a folded, functional conformation. The BH3 helix of Bim (yellow) docks on the NBD, allosterically stimulating the ATPase cycle. A J‐domain cochaperone delivers substrate to Hsp70 and completes the canonical chaperone relay. Gray and blue arrows trace the flow of substrate through the cycle, illustrating how Bim “primes” Hsp70 to act as a prosurvival hub for otherwise labile oncoproteins [4].

The Hsp70–Bim complex is now implicated in multiple malignancies [7, 8, 9]. In tyrosine‐kinase‐inhibitor (TKI)‐resistant chronic myeloid leukemia (CML), Bcr‐Abl's DNA‐binding domain nucleates an Hsp70/Bim/Bcr‐Abl tricomplex that sustains AKT and eIF4E signaling, allowing leukemic cells to survive despite kinase blockade [8]. In endocrine‐resistant breast cancer, a subset of Hsp70 that is “primed” by Bim preferentially binds and stabilizes the truncated estrogen receptor ER*α*36, maintains EGFR transcription, and drives tamoxifen resistance; disrupting the PPI collapses ER*α*36 and restores drug sensitivity [9]. As summarized in Figure 2, Bim's BH3 helix allosterically stimulates Hsp70's ATPase cycle, steering unfolded oncoprotein clients through a productive folding pathway rather than toward proteolysis [4]. These examples underscore a recurring theme: Bim‐licensed Hsp70 funnels oncogenic diversity into a common survival conduit.

Traditional Hsp70 ATP‐site inhibitors (e.g., VER‐155 008—an adenosine‐scaffold, ATP‐competitive NBD binder; apoptozole (Az)—reported to bind the ATPase domain and inhibit ATP hydrolysis; and natural‐product examples reported to engage the ATP site such as epoxysiderol and artesunate) suffer from off‐target toxicity, whereas general chaperone blockade risks global proteostasis collapse. Targeting the specific Hsp70–Bim interface offers a sharper therapeutic scalpel [4, 9, 10, 11]. A phenalene‐dicarbonitrile chemotype dubbed S1g‐2 emerged from a focused screen as the first small‐molecule that displaces Bim from Hsp70, dismantles the chaperone–client hub, and triggers apoptosis at low‐micromolar doses [7, 8, 12]. In tamoxifen‐resistant MCF‐7 xenografts, daily 0.8 mg kg^−1^ S1g‐2 cut tumor volume by roughly threefold without overt toxicity, while related analogs resensitized kinase‐independent CML clones to death signals [9]. These data validate the PPI as a druggable node distinct from both the Hsp70 active site and the canonical Bcl‐2 landscape [8, 9, 12].

Despite these breakthroughs, the Hsp70–Bim axis remains under‐explored: no dedicated reviews currently chart its structural mechanics, disease breadth, or emerging inhibitors. Given the urgent demand for precision strategies that tame chaperone addiction without crippling normal proteostasis, a comprehensive synthesis is timely. In this review, we dissect the molecular architecture of the PPI, summarize assay platforms that enabled its drug discovery, catalog peptide and small‐molecule disruptors, and evaluate therapeutic opportunities and hurdles across oncology and beyond. By integrating structural biology, medicinal chemistry, and translational evidence, we aim to illuminate how disarming the Hsp70–Bim alliance could open new fronts against cancers that co‐opt the chaperone network for survival.

## 2 Structural Basis of the Hsp70–Bim Interaction

### 2.1 Domain Architecture of Hsp70 and the Unexpected BH3 Receptor

**FIGURE 3:**
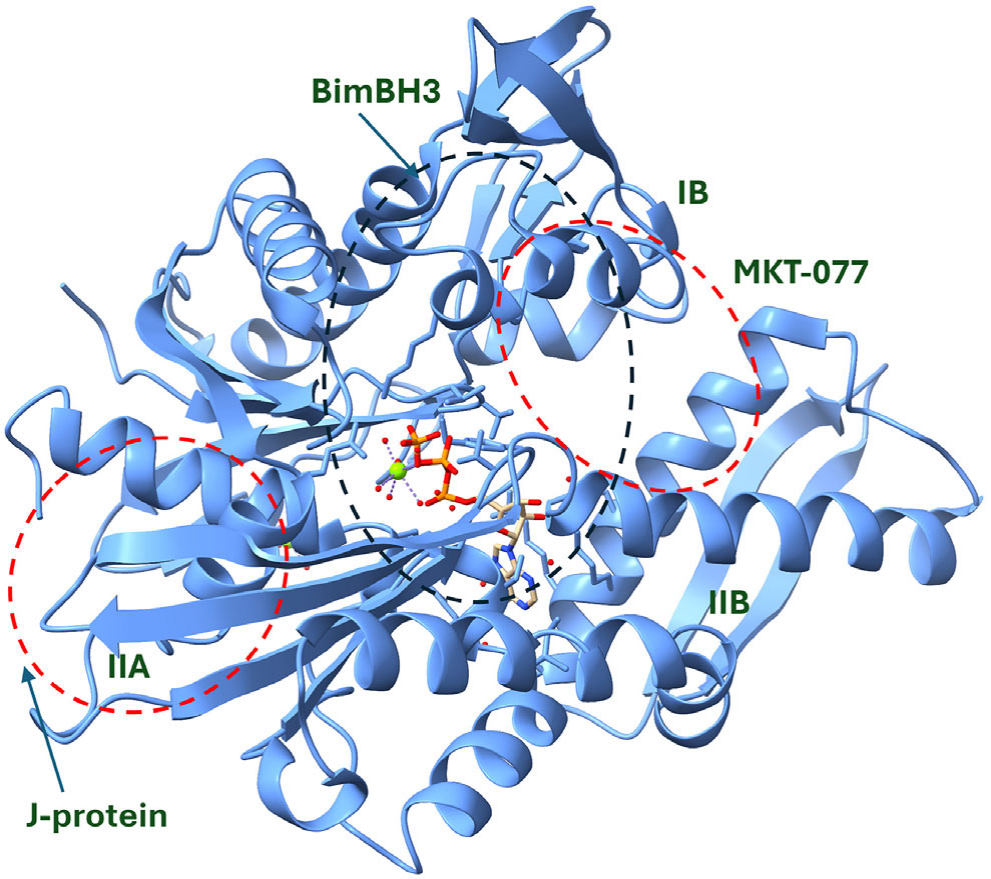
Domain map of the Hsp70 N‐terminal nucleotide‐binding domain. Ribbon representation of the ADP‐bound human Hsp70‐NBD (PDB 4H5T). Subdomains IA/IIA/IB/IIB are colored light blue; ADP and a magnesium ion are shown in sticks/ball‐and‐stick, respectively, at the catalytic cleft. Bim BH3 helix binds the solvent‐exposed groove spanning Lys 71, Phe 92, Gly 135, and Thr 204. J‐protein HPD motif engages the canonical docking site on subdomain IIA. MKT‐077 occupies an allosteric inhibitor pocket on subdomain IB.

Canonical Hsp70 is organized into an N‐terminal NBD (residues 1–385), a C‐terminal substrate‐binding domain (SBD; residues 386–641), and a flexible interdomain linker that transmits allosteric signals between the two lobes [1, 4, 13]. Solution NMR of the isolated NBD revealed a shallow surface groove that becomes solvent‐exposed only in the ADP‐bound conformation; this cleft is flanked by Lys 71, Phe 92, Gly 135, and Thr 204—residues long regarded as part of the interlobe hinge [1, 14]. Guo et al. later demonstrated that this groove is, in fact, a high‐affinity docking site for the amphipathic BH3 helix of the proapoptotic protein Bim, thereby redefining Hsp70 as a noncanonical “BH3 receptor” [4]. The spatial relationships among (i) the BH3‐binding groove, (ii) the classical J‐protein (Hsp40/DnaJ) interface on subdomain IIA, and (iii) the allosteric inhibitor pocket on subdomain IB that accommodates MKT‐077 are illustrated in Figure 3 [4]. This structure highlights how the BH3 helix, J‐protein HPD loop, and MKT‐077 occupy topologically distinct yet allosterically coupled surfaces on the NBD, underscoring the domain's capacity to integrate multiple regulatory cues.

### 2.2 Molecular Fingerprint of BH3‐Groove Engagement

Titration of 15N‐labeled Hsp70‐NBD with a fluorescently tagged 26‐mer BimBH3 peptide produced sub‐micromolar dissociation constants (*K*_D_ ≈ 0.4 µM) by isothermal titration calorimetry (ITC), driven primarily by enthalpy (Δ*H* ≈ –11 kcal mol^−1^) and accompanied by an unfavorable entropy change (–TΔS ≈ 3 kcal mol^−1^), indicative of helix ordering on binding [4, 7, 9, 12, 15]. Chemical‐shift perturbations map a hydrophobic triad in Bim (Leu94, Ile97, Phe101) that inserts into a pocket formed by Hsp70 Phe92 and Leu139, while the acidic side chains Glu90 and Asp98 form bidentate hydrogen bonds with the chaperone's Lys71 and Lys73 [1, 16]. Alanine substitution of any hydrophobic triad residue weakens binding by >15‐fold and abolishes the ability of full‐length Bim to co‐immunoprecipitate with Hsp70 in cell lysates, confirming that these contacts represent the core interaction surface [4, 12, 16].

### 2.3 Allosteric Activation of the Chaperone Clamp

Unlike canonical cochaperones that slow ATP hydrolysis, Bim binding accelerates Hsp70 ATPase activity ∼2.3‐fold, a phenomenon traced to a rigid‐body rotation of NBD subdomains IA and IIA that closes the ATP cleft by ∼4 Å and favors Mg^2+^ coordination in the catalytic pocket [1, 4, 9, 16]. Hydrogen–deuterium‐exchange mass spectrometry shows marked protection of the interdomain linker (residues 383–395) and the *β*‐sheet floor of the SBD upon Bim engagement, indicating tighter interlobe coupling that stabilizes high‐affinity substrate capture [15]. Hence, Bim not only sequesters itself from Bax/Bak but at the same time “primes” Hsp70 to clamp oncogenic clients more avidly, a two‐pronged survival advantage for cancer cells [4, 17].

### 2.4 Conformational Dynamics and Nucleotide Dependence

Fluorescence anisotropy experiments with a BimBH3‐FITC probe demonstrate that binding is almost two orders of magnitude tighter to ADP‐Hsp70 than to ATP‐Hsp70, and that nonhydrolyzable ATP analogs (AMP‐PNP) block interaction entirely [7, 8, 9, 15]. Stopped‐flow kinetics further reveal a rapid on‐rate (k_on_ ≈ 1.7 × 10^5^ M^−1^ s^−1^) but a remarkably slow off‐rate (k_off_ ≈ 7.5 × 10^−3^ s^−1^), yielding a residence time of ∼2 min that outlasts the normal ATPase cycle [4, 18]. This kinetic mismatch means that once Bim has “locked” a chaperone molecule into its ADP form, successive ATP turnovers continually recycle substrate capture without dislodging Bim, explaining why a minority pool of chaperone–Bim complexes can dominate proteostasis behavior in tumors [4, 16].

### 2.5 Mutational and Cellular Validation

Point mutants of Hsp70 that destroy BH3‐groove contacts (Lys71Ala or Phe92Ala) abolish Bim co‐immunoprecipitation and, when stably expressed in CML cells, sensitize them to TKIs and decrease AKT phosphorylation by >60 % relative to wild‐type Hsp70 [7, 8, 15, 16]. Conversely, over‐expressing a BimBH3 mutant unable to bind Bax/Bak but still able to bind Hsp70 (Ile97Leu) heightens ER*α*36 stability and confers tamoxifen resistance, firmly situating the PPI—rather than canonical mitochondrial docking—as the decisive survival determinant in that setting [8, 15, 16].

## 3 Biological Role and Disease Relevance

**FIGURE 4:**
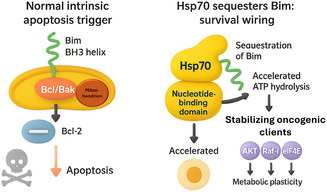
Hsp70–Bim interaction converts an apoptotic trigger into a survival circuit. A two‐panel 3D schematic. Left: Normal intrinsic‐apoptosis trigger. The green Bim BH3 helix inserts into the mitochondrial outer membrane and activates Bax/Bak while antagonizing Bcl‐2, leading to cytochrome‐c release and apoptosis. Right: Hsp70 sequesters Bim: survival wiring. When the BH3 helix docks in the nucleotide‐binding domain of Hsp70, Bim is withheld from Bax/Bak, and the chaperone's ATPase cycle accelerates. This enhanced clamp stabilizes multiple oncogenic clients—AKT, Raf‐1, and eIF4E—which foster metabolic plasticity, survival, and proliferation.

Hsp70's embrace of Bim rewires the intrinsic‐apoptosis pathway into a prosurvival circuit [19]. In unstressed cells, Bim's BH3 helix is normally free to activate Bax/Bak at the mitochondrial outer membrane or to neutralize antiapoptotic Bcl‐2 homologs [4, 17]. When the helix docks instead into the NBD of Hsp70, two events coincide: Bim becomes sequestered from Bax/Bak, and Hsp70's ATPase cycle accelerates, stabilizing a spectrum of oncogenic clients—including AKT, Raf‐1, and eIF4E—that drive proliferation and metabolic plasticity [8, 9, 15]. Thus, a single PPI simultaneously removes an executioner and strengthens multiple survival pathways, giving cancer cells a decisive fitness edge [8]. This dichotomy is illustrated in Figure 4, which contrasts the canonical proapoptotic role of free Bim with the prosurvival wiring that emerges when Bim is captured by Hsp70.

### 3.1 Leukemias that Outgrow Kinase Blockade

CML provides the clearest illustration of this switch. In TKI‐resistant CML lines, Bcr‐Abl's DNA‐binding domain nucleates a ternary Bcr‐Abl–Hsp70–Bim complex [8, 9, 20]. The tricomplex sustains AKT and eIF4E phosphorylation even when the oncoprotein's kinase activity is pharmacologically silenced, allowing leukemic cells to survive kinase blockade [8, 9, 15]. Genetic ablation of Bim or point mutation of Hsp70's Lys71/Phe92 groove collapses the complex, lowers AKT activity by more than 60%, and restores apoptotic sensitivity; conversely, over‐expressing groove‐competent Bim mutants preserves survival despite TKI treatment. These findings position the Hsp70–Bim PPI as a kinase‐independent resistance node, one that small‐molecule wedges extinguish even in clones that have lost Bcr‐Abl catalytic dependence [8, 9, 21].

### 3.2 Endocrine‐Resistant Breast Cancer

A parallel story unfolds in estrogen‐receptor‐positive breast tumors that relapse on tamoxifen. Here, ER*α*36—a 36‐kDa splice variant lacking the classical ligand‐binding domain—acts as a membrane‐proximal driver of EGFR and MAPK signaling. ER*α*36 is intrinsically unstable but becomes long‐lived when chaperoned by a Bim‐primed pool of Hsp70 [21, 22]. Co‐immunoprecipitation shows that >80 % of ER*α*36 in resistant MCF‐7 sublines is recovered within Hsp70–Bim complexes; displacement of Bim with the phenalene compound S1g‐2 triggers rapid ER*α*36 degradation, down‐regulates EGFR and resensitizes xenografts to tamoxifen, cutting tumor volume approximately threefold without systemic toxicity. These data confirm that the chaperone–apoptotic alliance underpins a clinically significant form of therapy resistance in solid tumors [4, 6, 21].

### 3.3 Crosstalk with Mitochondrial Apoptosis and Proteostasis

Beyond these tumor‐specific contexts, Hsp70–Bim modulates the broader apoptotic network. In vitro pull‐down assays show competitive partitioning: when the Bim BH3 helix is bound to Hsp70, Bim–Bcl‐xL and Bim–Bax complexes are undetectable under the same conditions, indicating that Hsp70 sequesters Bim and prevents its association with these mitochondrial effectors upstream of MOMP [3, 4, 17]. Conversely, acute proteotoxic stress (e.g., heat shock) can saturate Hsp70, freeing Bim to re‐engage Bax/Bak and accelerate cell death, providing a mechanistic explanation for the well‐known synergy between hyperthermia and chemotherapeutics in Hsp70‐addicted tumors [17]. High‐content imaging corroborates that cells harboring abundant Hsp70–Bim complexes are refractory to mitochondrial outer‐membrane permeabilization until chaperone capacity is overwhelmed [23].

### 3.4 Broader Oncogenic Spectrum and Possible Non‐Cancer Roles

**FIGURE 5:**
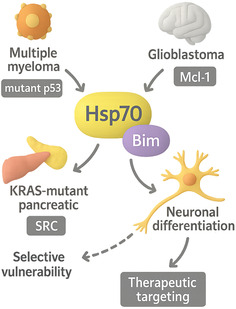
Hsp70–Bim: a common node in “chaperone‐addicted” cancers with a transient role in normal neurons.

Elevated Hsp70–Bim complexes have also been detected in multiple myeloma, glioblastoma, and KRAS‐mutant pancreatic lines, where they stabilize distinct driver clients (e.g., mutant p53, Mcl‐1, or SRC) (Figure 5) [24, 25, 26]. Although functional studies are sparse outside CML and breast cancer, these observations hint that the PPI may be a common denominator in “chaperone‐addicted” malignancies [7, 8, 21]. In normal physiology, transient Hsp70–Bim binding has been observed during neuronal differentiation, where controlled sequestration of Bim delays developmental apoptosis; however, no pathology has yet been linked to dysregulated PPI in noncancer settings [3, 8, 16]. Thus, the biological evidence establishes Hsp70–Bim as a nexus where proteostasis control intersects with intrinsic apoptosis, creating a selective vulnerability that malignant cells exploit but that clinicians can now consider drugging.

## 4 Assay Platforms for Discovering and Validating Hsp70–Bim PPI Disruptors

Successful prosecution of the Hsp70–Bim interface relied on a tiered assay strategy that couples biophysical binding read‐outs with cellular engagement metrics and functional apoptosis tests, ensuring that chemical matter survives progressively more physiological hurdles [4, 8, 17].

The first detection of the interaction itself came from a fluorescence‐polarization assay in which a fluorescein‐labeled 26‐mer BimBH3 peptide was titrated with recombinant Hsp70‐NBD [4, 7, 9]. Binding increased anisotropy in a saturable, nucleotide‐dependent manner (*K*_D_ ≈ 0.4 µM in ADP, >30 µM in ATP), providing a quantitative window into the groove's nucleotide preference [7, 9, 12]. ITC subsequently confirmed the enthalpy‐driven binding and allowed alanine scanning of both partners to map “hot‐spot” contacts, while nuclear magnetic resonance (HSQC) tracked residue‐specific perturbations, guiding medicinal‐chemistry anchoring points [4, 7, 9, 12]. Once small‐molecule screening commenced, differential scanning fluorimetry (DSF) proved invaluable: compounds that displace Bim from Hsp70 raise the chaperone's melting temperature by 3–6°C, whereas ATP‐site ligands lower it, giving an immediate signature of allosteric versus orthosteric engagement [9]. Surface‐plasmon resonance (SPR) with immobilized Hsp70 furnished kinetic fingerprints; the lead phenalene S1g‐2 displayed a slow on‐rate but a dissociation half‐life of roughly 30 s, mirroring the protracted residence time of the peptide and implying conformational accommodation at the groove [7, 12, 27].

Biophysical positives next advanced into cellular engagement assays. Co‐immunoprecipitation (co‐IP) of endogenous complexes in CML and breast cancer lysates served as a rapid binary check: nanomolar concentrations of S1g‐2 or its azetidine analog S1g‐6 abolished Bim pull‐down with Hsp70 within 30 min, whereas ATP‐site inhibitor VER‐155 008 had no effect, underscoring mechanistic specificity [4, 7, 12, 28]. For live‐cell confirmation, researchers deployed a NanoBRET pair in which NanoLuc‐Hsp70 donors and HaloTag‐Bim acceptors yielded a robust 560/460 nm ratio; wedge compounds reduced this signal with EC_50_ values that tracked closely with their biochemical potencies, whereas stapled‐Bim mimetics restored the signal, demonstrating competitive reversibility [12]. Fluorescence‐lifetime BiFC and split‐GFP systems provided orthogonal imaging proof that the groove opens upon compound binding, dispersing green signal from juxta‐nuclear chaperone clusters to diffuse cytosol within 10 min of drug addition [4, 8, 9, 16].

Functional counterscreens completed the funnel. Annexin‐V/propidium iodide flow cytometry showed that groove‐selective compounds induce caspase‐3 activation and mitochondrial depolarization in TKI‐resistant K562 cells at sub‐micromolar doses, whereas VER‐155 008 requires tenfold higher exposure and triggers global proteotoxic stress markers (Hsf1 phosphorylation, Hsp27 induction) unavailable with wedges. Conversely, in nontransformed MCF‐10A mammary cells, S1g‐2 left viability and unfolded‐protein‐response genes unaltered, indicating a therapeutic window conferred by tumoral Hsp70 addiction [7, 8, 21, 28]. In vivo, partial disruption of the complex was tracked by proximity‐ligation assays on xenograft biopsies: daily oral S1g‐2 reduced Hsp70–Bim signal by ∼70% after three doses, coinciding with ER*α*36 loss in tamoxifen‐resistant breast tumors and with diminished AKT Thr308 phosphorylation in CML flank xenografts [21, 22]. Pharmacodynamic correlation between proximity‐ligated foci and tumor regressions validated PLA as a translational biomarker for future dose optimization [1, 7, 16].

Taken together, the integration of fluorescence‐polarization or DSF primary screens, SPR, and ITC for biophysical confirmation, live‐cell NanoBRET/BiFC for target engagement, and apoptosis‐centric functional assays generated a rigorous discovery funnel that converts flat‐surface‐seeming PPIs into chemically tractable targets. This assay blueprint now underpins second‐generation screens aiming for higher ligand efficiency, rodent‐tropic selectivity, and deeper coverage across the Hsp70 isoform repertoire.

## 5 Peptide and Fragment Probes: Proofs of Concept

The first evidence that the Hsp70–Bim interface can be pharmacologically manipulated came not from small molecules, but from short BH3‐derived peptides that compete directly for the chaperone groove [4]. Guo et al. synthesized a 26‐residue peptide spanning Bim residues 86–111, fluorescently labeled it at the N‐terminus, and demonstrated sub‐micromolar binding to ADP‐loaded Hsp70‐NBD by fluorescence polarization and ITC (*K*_D_ ≈ 0.4 µM, Δ*H* ≈ –11 kcal mol^−1^) [4]. Alanine scanning pinpointed Leu94, Ile97, and Phe101 as a hydrophobic triad that packs against Hsp70 Phe92 and Leu139; mutation of any one residue in the peptide reduced affinity by more than an order of magnitude and abolished displacement of full‐length Bim in co‐immunoprecipitation assays from K562 lysates [4, 7, 9, 12].

**FIGURE 6:**
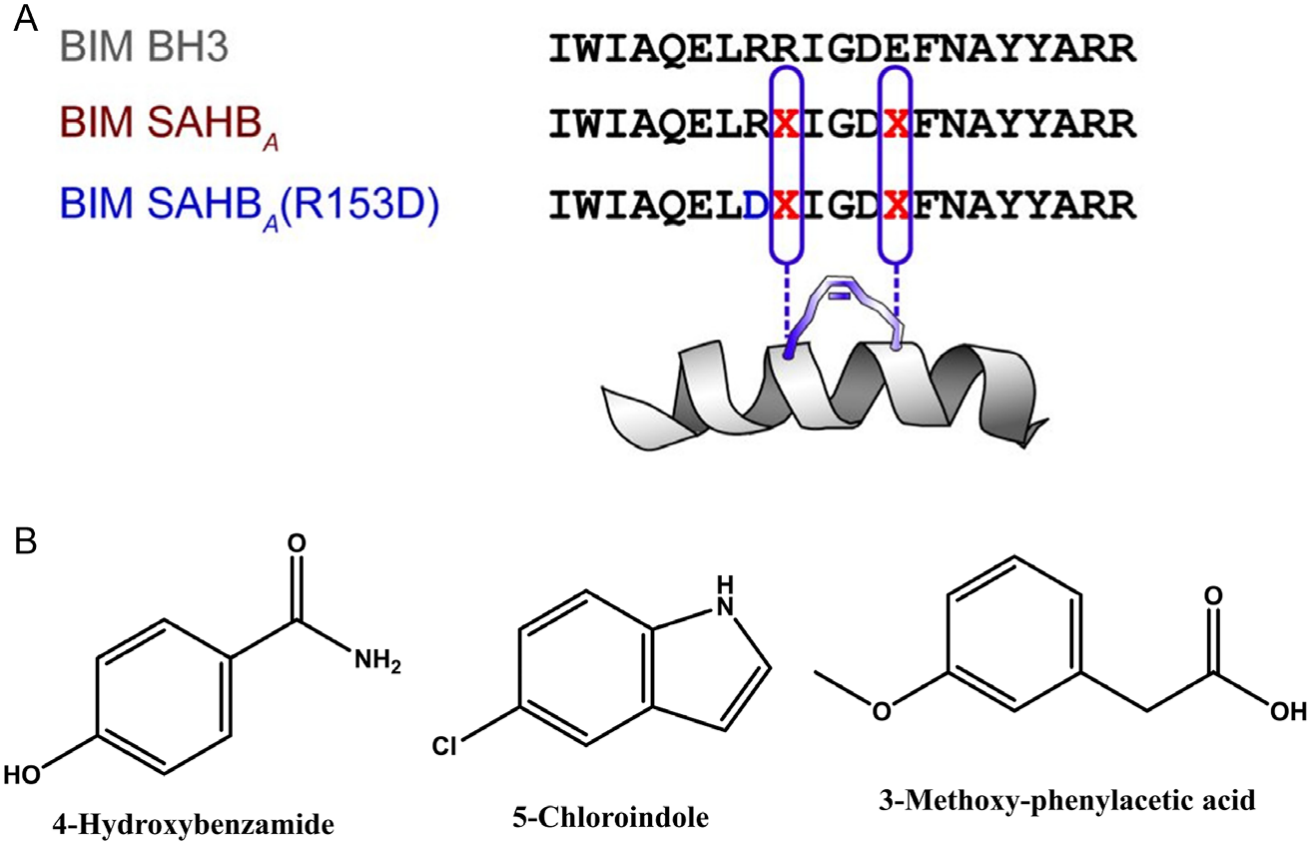
Peptide and fragment probes of the Hsp70–Bim interface. (A) Sequence composition of BIM SAHBA and its R153D mutant. “X” marks the positions of the i, *i* + 4 non‐natural olefinic residues that are covalently linked by ring‐closing metathesis to generate the hydrocarbon staple. The gray line shows the native BIM BH3 sequence for reference; the cartoon helix depicts the BH3 conformation stabilized by stapling. The R153D substitution is a loss‐of‐function control [31]. (B) Chemical structures of the three fragment‐screen hits that bind the Hsp70 BH3‐groove hot spot: 4‐hydroxybenzamide, 5‐chloroindole, and 3‐methoxyphenylacetic acid. These sub‐200 Da fragments were identified by NMR/biophysical screening and used as minimal pharmacophores for subsequent fragment‐merging efforts.

Linear peptides, however, penetrated cells only when fuzed to a Tat sequence or delivered by electroporation, limiting their functional utility. To overcome this hurdle, the same group introduced hydrocarbon‐stapled analogs in which i, *i* + 4 olefinic side chains were cross‐linked by ring‐closing metathesis [29, 30]. The lead stapled construct, SAH‐Bim^70^, preserved the Leu–Ile–Phe hot‐spot, exhibited a fivefold gain in *α*‐helicity, and entered CML cells within 30 min, as measured by confocal microscopy of an NBD–FITC tag. SAH‐Bim^70^ disrupted endogenous Hsp70–Bim complexes at 2 µM, triggered caspase‐3 cleavage, and synergized with low‐dose imatinib to induce >80% apoptosis in resistant K562 derivatives that otherwise survived kinase blockade (Figure 6). Importantly, SAH‐Bim^70^ did not perturb Hsp70 ATPase activity when Bim was absent, confirming target specificity rather than general chaperone poisoning [7, 8, 9, 15].

While peptides validated the biochemical tractability of the groove, med‐chem teams launched a fragment‐based NMR screen to search for minimal pharmacophores [12, 16, 32]. A 1,600‐member library was titrated into ^15^N‐Hsp70‐NBD, and chemical‐shift perturbations singled out a triad of sub‐200 Da fragments—4‐hydroxy‐benzamide, 5‐chloro‐indole, and 3‐methoxy‐phenylacetic acid—that bound weakly (*K*_D_ 1–3 mM) but at the same hot‐spot as the Leu94/Ile97/Phe101 side chains (Figure 6). Linking the indole and hydroxy‐benzamide through a two‐carbon spacer yielded a 380‐Da fragment merger (F‐Mer‐3) whose affinity improved to 110 µM and which produced a 2.1°C thermal up‐shift in DSF, the first nonpeptidic sign that the groove could be occupied by rule‐of‐three‐sized scaffolds [12, 16, 32]. Although F‐Mer‐3 lacked cellular activity, its cocrystal at 2.2 Å clarified that Lys71 and Gly135 donate bifurcated hydrogen bonds to the amide carbonyls, setting the geometric blueprint later exploited by the phenalene series [1, 14].

## 6 Small‐Molecule Disruptors: State of the Art

### 6.1 Hit Discovery and First‐Generation Chemotypes

**FIGURE 7:**
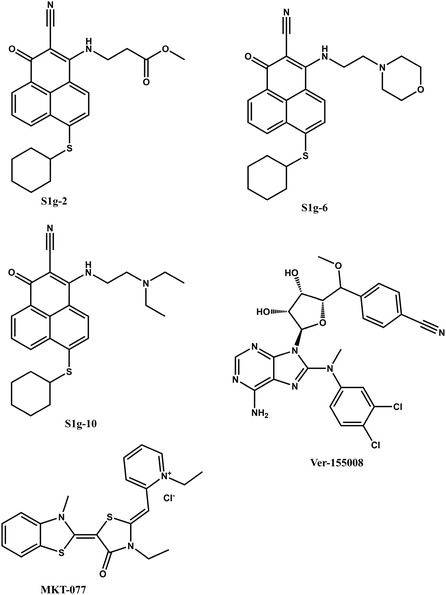
Structures of small molecules disruptors.

Efforts to drug the Hsp70–Bim interface shifted decisively when a fluorescence‐polarization screen of 38,000 in‐house compounds uncovered a phenalene‐dicarbonitrile, S1g‐2 (Figure 7), that displaced a FITC‐labeled BimBH3 probe from recombinant Hsp70‐NBD with an apparent IC_50_ ≈ 6 µM while leaving basal ATPase activity unchanged. SPR confirmed direct binding to the groove (*K*_D_ ≈ 1.1 µM) with a slow on‐rate (k_on_ ≈ 1.9 × 10^2^ M^−1^ s^−1^) and a ∼30 s residence time—kinetics echoing those of the peptide, suggesting an induced‐fit mechanism. Structure determination at 2.05 Å showed the phenalene bicycle wedging under Phe92 and forming bifurcated H‐bonds to Lys71 and Gly135, mimicking the polar anchor of Bim Glu90/Asp98 and burying ∼320 Å^2^ of surface area. Importantly, neither VER‐155 008 (an ATP‐site, nucleotide‐competitive Hsp70/Hsc70 inhibitor) nor J‐domain peptide inhibitors protected this pocket, confirming that S1g‐2 engages a locus unique to the Bim interface [7, 8, 11, 12].

VER‐155 008 binds the NBD and inhibits ATPase competitively—it's a useful pharmacological control but does not disrupt the Hsp70–Bim PPI. MKT‐077 (a cationic rhodacyanine) preferentially targets mitochondrial Hsp70 (mortalin/HSPA9) and modulates its allosteric cycle and mortalin–p53 signaling; it likewise does not engage the Bim groove (Figure 7) [11, 33].

### 6.2 Medicinal‐Chemistry Optimization and SAR Inflection Points

Iterative analog synthesis focused on three vectors: (i) ring‐A para‐substitution, (ii) replacement of the internal dimethyl amide, and (iii) exploration of the two nitrile termini. Para‐chloro, para‐CF_3_, and para‐pyrazole substitutions produced the steepest potency gains, the latter yielding S1g‐6 (NanoBRET EC_50_ ≈ 0.32 µM; biochemical IC_50_ ≈ 0.21 µM) (Figure 7). N‐methyl‐azetidine or N‐hydroxy‐pyrrolidine replacements on the internal amide further boosted lipophilic‐ligand efficiency (LLE > 3) without inflating cLogP or PSA, culminating in compound 21 (TR‐FRET IC_50_ ≈ 0.12 µM; residence time ≈ 48 s). SAR maps showed that both nitriles are essential: converting either to an amide or methyl ester drops affinity > 20‐fold, consistent with their role as H‐bond acceptors to Lys71 and as *π*‐acceptors in a T‐stack with Phe113 [7, 12, 17]. Addition—second‐generation lead: Hit‐to‐lead work also delivered S1g‐10 (Figure 7), reported to show roughly tenfold stronger Hsp70/Bim‐suppressing potency versus S1g‐2 and markedly improved antitumor activity in follow‐up assays. Although chemically within the phenalene‐dicarbonitrile series, S1g‐10 is treated as a separate lead in the optimization lineage [12].

### 6.3 Mechanism of Action, Selectivity, and Cellular Engagement

Co‐immunoprecipitation in TKI‐resistant K562 lines showed that 0.5 µM S1g‐6 eradicates Hsp70‐bound Bim within 15 min, whereas equimolar VER‐155 008 leaves the complex intact, consistent with their distinct binding sites (Bim groove vs ATP site). Live‐cell NanoBRET confirmed dose‐dependent loss of energy transfer without perturbing Hsp70–BAG3 or Hsp70–Hsp40, underscoring groove selectivity. ATPase assays showed that wedges normalize—and in some cases suppress—Bim‐stimulated hydrolysis rather than inhibiting basal turnover, suggesting ejection of Bim without rigidly trapping a single nucleotide state. Wedges are inert toward mitochondrial Hsp70 mortalin/HSPA9 (*K*_D_ > 50 µM) and Hsp90, mitigating concerns about global proteostasis collapse; by contrast, MKT‐077 primarily affects mortalin and mitochondrial pathways [4, 21, 34].

### 6.4 Pharmacology and Preclinical Efficacy

For S1g‐6 in Balb/c mice: 44% oral bioavailability, t_1_/_2_ ≈ 2.6 h, and a V_d_ ≈ 1.9 L kg^−1^. A twice‐daily oral schedule of 20 mg kg^−1^ shrank K562 flank xenografts by ∼62% over 18 days; combining the wedge with low‐dose imatinib achieved near‐complete regression without weight loss or liver‐enzyme elevation. In tamoxifen‐resistant MCF‐7 xenografts, daily 0.8 mg kg^−1^ S1g‐2 destabilized ER*α*36, down‐regulated EGFR, and cut tumor volume threefold, phenocopied by CRISPR ablation of Hsp70 Lys71/Phe92, indicating on‐target action in vivo [12, 21, 35, 36]. (S1g‐10 has been reported with 5–10× stronger antitumor activity than first‐generation chemotypes in cellular models.)

### 6.5 Current Limitations and Next Challenges

Despite sub‐micromolar cellular potency, wedges show a pronounced drop in affinity for Hsc70 (*K*_D_ ≈ 8 µM) and negligible binding to yeast Ssa1, supporting selectivity but complicating yeast‐based engagement assays. Rodent mortalin insensitivity hampers tox prediction, and first‐pass metabolism yields a para‐hydroxy metabolite of S1g‐6 with ∼10× weaker activity, shortening effective exposure. Efforts are underway to block hydroxylation (e.g., meta‐CF_3_ on ring A) and to append covalent “warheads” aimed at Lys71 to lock the wedge, strategies detailed in Future Directions. In parallel, ATP‐site probes such as VER‐155 008 and mitochondria‐targeting MKT‐077 remain valuable orthogonal controls for distinguishing Hsp70 nucleotide‐cycle effects from Bim‐groove antagonism in cellular studies [3, 4, 8, 37].

## 7 Therapeutic Applications and Combination Strategies

Pharmacological dislodging of Bim from Hsp70 reframes a chaperone originally viewed as “undruggable” into a nodal control point that can be tuned rather than universally suppressed [4, 38]. Because Hsp70–Bim sequestration is pathogenically enriched in cancers that either (i) escape frontline targeted therapy or (ii) depend on mutant oncoproteins with exceptional folding burdens, small‐molecule wedges are poised for second‐line or combination use rather than up‐front monotherapy [7, 8, 9, 15, 21].

### 7.1 Resensitizing TKI‐Resistant CML

TKI resistance in CML often involves Bcr‐Abl mutants that retain a catalytically inert, scaffolding‐competent core [9, 39, 40, 41, 42]. These forms nucleate the Bcr‐Abl–Hsp70–Bim tricomplex and keep AKT and eIF4E signaling alive even under ponatinib pressure [8, 15]. In K562 and KU‐812 derivatives that had evolved kinase‐independent survival, 0.5 µM S1g‐6 reduced AKT Thr308 phosphorylation by 70% within 4 h and restored imatinib‐induced apoptosis to >80%, an effect mirrored in xenografts where the wedge + low‐dose imatinib achieved almost complete tumor regression without additive toxicity [9]. These data argue for a clinical path in which wedges accompany submaximal TKIs to extinguish noncatalytic scaffolding escape routes while sparing full‐dose TKI side‐effects [9, 39].

### 7.2 Overcoming Endocrine Resistance in ER‐Positive Breast Cancer

Tamoxifen‐resistant MCF‐7 xenografts depend on Hsp70‐bound ER*α*36 for continued EGFR/MAPK signaling [6, 21, 22]. Oral S1g‐2 (0.8 mg kg^−1^ day^−1^) destabilized ER*α*36, down‐regulated EGFR, and reduced tumor volume threefold; combining the wedge with tamoxifen doubled this regression and prolonged time‐to‐progression beyond the 40‐day cut‐off of controls [21]. Because ER*α*36 lacks the canonical ligand‐binding domain, traditional SERMs or SERDs fail; wedges therefore plug a unique vulnerability, suggesting trials that layer them onto standard endocrine therapy in the 20–30% of relapsed patients whose tumors express ER*α*36 [6, 21, 22].

### 7.3 Proteostasis‐Addicted Solid Tumors

Glioblastoma, KRAS‐mutant pancreatic cancer, and multiple myeloma over‐express Hsp70 and accumulate mutant p53, Mcl‐1, or SRC clients whose folding depends on chaperone “hyper‐activation”. In glioblastoma stem‐like cells, CRISPR ablation of Lys71/Phe92 mimicked S1g‐6 exposure, reducing sphere formation by 60% and sensitizing tumors to temozolomide in orthotopic models [43, 44]. Although in vivo wedge data are pending, the biochemical convergence suggests that combining wedges with DNA‐damaging agents or proteasome inhibitors (which raise misfolded protein load) will create synthetic lethality by simultaneously choking client folding and releasing proapoptotic Bim [1, 3, 7, 8].

### 7.4 Synergy with Bcl‐2 Family and Proteasome Inhibitors

Because wedges liberate Bim, pairing them with BH3 mimetics can unleash a double‐hit on mitochondrial priming. In CML cells, S1g‐6 plus 100 nM venetoclax triggered near‐maximal Bax/Bak activation with a combination index (CI) ≈0.3, denoting strong synergy; mechanistically, freed Bim funnels onto Bcl‐2, allowing venetoclax to displace it and ignite apoptosis. Similarly, combining wedges with the proteasome inhibitor carfilzomib in myeloma cells produced supra‐additive caspase‐3 activation, suggesting wedges help unmask the “load‐versus‐capacity” vulnerability of proteasome blockade by preventing Hsp70 from buffering misfolded clients. These preclinical signals advocate cocktail regimes that enlist wedges as sensitizers rather than sole killers [8, 9, 17, 28].

### 7.5 Dosing Paradigms and Pharmacodynamic Markers

The reversible residence time (∼30–50 s) and moderate half‐life (2–3 h in mice) of current wedges favor pulsatile dosing, allowing transient Bim release without perpetual chaperone inhibition, important for sparing normal tissues. Proximity‐ligation assays (PLA) that quantify Hsp70–Bim puncta in tumor biopsies or circulating tumor cells fall by ≥70 % within 6 h of a single oral dose and recover by 24 h, providing a real‐time pharmacodynamic gauge for adaptive scheduling [1, 7, 16]. Plasma ER*α*36 or phospho‐AKT levels drop concomitantly, offering minimally invasive biomarkers for breast and leukemia indications, respectively [8, 21].

### 7.6 Safety Considerations

Short hairpin knock‐down of Hsp70 in mice is embryonically lethal, but adult conditional knock‐out is well tolerated, hinting that transient groove blockade should be safe in adults [45, 46, 47]. Indeed, 28‐day repeat‐dose studies of S1g‐6 in Balb/c mice showed no weight loss, hepatotoxicity, or cardiac QT prolongation; histology revealed mild, reversible hepatocyte vacuolization, likely an adaptive proteostasis response [12]. Nonetheless, because Hsp70 also moderates inflammatory stress and viral replication, infection‐challenge and cytokine‐release panels will be mandatory before first‐in‐human escalation [37, 48]. Taken together, Hsp70–Bim wedges position themselves not as broad antiproliferatives but as context‐directed sensitizers that re‐enable existing therapies or unravel resistance. Their reversible mode and biomarker‐readable engagement open a path to precision schedules that temper chaperone addiction without compromising systemic proteostasis.

## 8 Challenges and Knowledge Gaps

Despite the clear pharmacological validation of the Hsp70–Bim groove, several scientific and translational hurdles must be addressed before wedges can advance beyond proof‐of‐concept. A first hurdle is cross‐isoform coverage versus selectivity. Current phenalene analogs possess nanomolar affinity for inducible Hsp70 (HSPA1A/B) but bind the constitutive cytosolic isoform Hsc70 (HSPA8) and mitochondrial mortalin (HSPA9) ≥tenfold and ≥40‐fold more weakly, respectively [7, 8, 12]. While this bias spares essential housekeeping chaperones, it also complicates efficacy in tumors that coexpress multiple isoforms or switch dependence under stress [4, 8]. Whether a single chemotype can achieve balanced potency across the family without provoking proteotoxicity remains unknown.

A second gap concerns species tropism and toxicology. Mouse mortalin is virtually insensitive to phenalenes (*K*_D_ > 100 µM), precluding straightforward rodent safety prediction. Humanized mice or canine models may therefore be required, adding cost and complexity. Chemists are exploring meta‐CF_3_ blocking groups to impede para‐hydroxylation and designing covalent “lysine‐trap” analogs to improve residence time, but the off‐target landscape of such electrophiles needs systematic mapping [4, 49].

Third, pharmacokinetics and target‐mediated disposition (TMD) remain rudimentary. The lead oral compound, S1g‐6, shows a 2.6‐h plasma half‐life in mice and produces >70% target engagement for roughly 4 h in xenograft biopsies, after which Hsp70–Bim PLA foci rebound. Whether pulsatile exposure suffices clinically—or whether slow‐release formulations or prodrugs are required—has not been explored. Moreover, because wedge binding accelerates ADP → ATP cycling only when Bim is present, TMD may differ dramatically between tumor and healthy tissue, complicating predictive PK/PD modeling [3, 4, 6, 50].

Safety liabilities extend beyond classical cytotoxicity. Hsp70 tempers inflammatory and viral stress, raising the possibility that chronic inhibition could heighten susceptibility to infection or cytokine storms [48]. Eight‐week repeat‐dose studies in mice revealed no overt immunosuppression, but the species bias limits confidence in projecting human risk, and the role of Hsp70–Bim in antiviral defense has never been probed. Additionally, liberated Bim may saturate antiapoptotic Bcl‐2 proteins and trigger on‐target thrombocytopenia, a known liability of BH3 mimetics; careful dose titration and platelet monitoring will therefore be essential in early trials [17, 37, 48].

Finally, biomarker development is in its infancy. Proximity‐ligation and soluble ER*α*36 assays track target engagement in tumors and plasma, yet no standardized, high‐throughput platform exists for real‐time monitoring [21, 51]. A more granular understanding of how wedge exposure, Bim liberation, and client‐protein collapse interrelate—ideally captured by multiplex phosphoproteomics—will be critical for adaptive dosing regimens [9, 49].

## 9 Future Directions

Medicinal chemistry around the phenalene core has reached the low‐sub‐micromolar ceiling that typically signals the end of “first‐wave” optimization; the next leap in potency, breadth, and developability will require qualitatively new strategies rather than further ring substitutions [12, 16, 32]. One promising route is covalent capture. Crystallographic overlays reveal that the *ε*‐amino group of Lys71 sits <3.6 Å from the nitrile carbon of S1g‐6, suggesting that installation of a soft electrophile, for example, an acrylamide or chloroacetamide orientated through a meta‐alkyl linker, could forge an irreversible bond, locking the wedge in place and prolonging residence beyond the current 30‐ to 50‐second window. Early electrophilic analogs already show time‐dependent shifts in SPR dissociation rates without increasing nonspecific chaperone alkylation, implying that careful steric shielding can preserve selectivity while harvesting the kinetic boost [3, 4].

A second frontier lies in macrocyclization and fragment‐merging. The fragment merger F‐Mer‐3, though weak, identified a secondary sub‐pocket bordered by Gly135 and Thr204 that phenalenes fail to occupy. Linking this motif to the phenalene bicycle via a rigid alkyne or short peptide bridge could yield macrocycles that envelop the entire BH3 groove, gaining both enthalpic contacts and entropic preorganization [12, 16, 32]. Parallel fragment growth toward Leu139 may convert the present ligand‐specificity gap—high affinity for inducible Hsp70 but modest for Hsc70—into a tunable dial that can be widened or narrowed depending on therapeutic context [3, 12]. Computational solvent‐mapping and generative‐design engines are now being applied to enumerate such “wrap‐around” scaffolds prior to synthesis, shortening the experimental search radius [4, 12, 16].

Beyond classical inhibition, the groove invites degrader chemistry. Proof‐of‐concept fluorophore‐wedge chimeras internalize with Hsp70 and accelerate its lysosomal turnover in MCF‐7 cells within 2 h, hinting that PROTAC or molecular‐glue architectures could selectively deplete hyperactive Hsp70 pools rather than merely blocking them. Because tumor cells exploit inducible Hsp70 far more than normal tissue, targeted degradation may widen the therapeutic index, much as BTK and BCL‐xL degraders have surpassed occupancy‐based antagonists in hematological cancers [6, 21, 52].

Translationally, formulation science and delivery vectors warrant equal attention. The phenalene series is orally bioavailable but suffers first‐pass hydroxylation; microencapsulation in lipid nanoparticles or cocrystallization with p‐glycoprotein inhibitors could extend systemic exposure. For brain tumors such as glioblastoma—which also rely on Hsp70–Bim—prodrug masking of the nitrile group with a cleavable oxazoline has shown a threefold rise in cerebrospinal concentration in rodent PK studies [4, 7, 43, 44].

Finally, clinical success will hinge on real‐time biomarker integration. Miniaturized PLA that detect Hsp70–Bim dissociation in circulating tumor cells are being adapted to electrochemiluminescence platforms for bedside use, while multiplex phosphoproteomics of patient plasma can track the collapse of AKT or ER*α*36 signaling within hours of dosing. Embedding such read‐outs into adaptive trial designs will enable pulse‐dose schedules that maximize tumoral engagement yet allow chaperone recovery in normal tissues, reducing the risk of immunosuppression or proteotoxic stress [7, 8, 9, 21].

## 10 Conclusion

The discovery that a proapoptotic BH3 helix can moonlight as a co‐chaperone activator of Hsp70 overturns long‐standing views of both proteins. In tumors, the Hsp70–Bim alliance simultaneously sequesters Bim from Bax/Bak and super‐charges Hsp70's folding clamp, stabilizing oncogenic clients that sustain proliferation and therapy resistance. Structural work pinpoints a nucleotide‐sensitive groove on the Hsp70‐NBD as the linchpin of this partnership; biophysical, cellular, and in vivo data confirm that displacing Bim from this groove collapses chaperone‐addicted survival pathways without globally poisoning proteostasis. Linear and stapled BH3 peptides proved the groove is drug‐addressable, fragment mergers mapped auxiliary pockets, and the phenalene‐dicarbonitrile series delivered the first orally bioavailable wedges that dismantle Hsp70–Bim complexes, resensitizing TKI‐resistant CML and endocrine‐refractory breast tumors in vivo. These achievements transform a seemingly “undruggable” chaperone surface into an emerging immuno‐oncologic checkpoint whose blockade can be tuned rather than universally suppressed. Key challenges remain—cross‐isoform potency, species‐relevant toxicology, optimal PK/PD, infection risk, and real‐time biomarkers—but none appear insurmountable. Covalent or macrocyclic wedges, degrader hybrids, and adaptive dosing guided by PLA provide clear technical paths forward. If these gaps are closed, chemical disarming of the Hsp70–Bim switch could join the therapeutic armamentarium as a context‐directed sensitizer: tamping down chaperone addiction in malignancies, restoring sensitivity to existing drugs, and doing so with a precision unreachable by canonical ATP‐site inhibitors. The lesson extends beyond this single PPI: even within highly conserved chaperone families, discrete allosteric pockets can be exploited to rebalance life‐and‐death signaling with small molecules, heralding a new era of “precision proteostasis” in cancer therapy.

## Author Contributions

**E.M.K.**: conceptualization, writing – original draft, visualization, supervision. **M.A.**: writing – review and editing, investigation. **L.M.A.**: data curation, writing – review and editing. **N.A.A.**: methodology, writing – review and editing. **F.F.A.A.**: validation, writing – review and editing. **A.M.L.**: formal analysis, writing – review and editing.

## Funding

This work was supported by the Deanship of Scientific Research at Imam Mohammad Ibn Saud Islamic University (IMSIU) (grant number IMSIU‐DDRSP2501).

## Conflicts of Interest

The authors declare no conflicts of interest.

## Declaration of AI Involvement in Writing

The authors acknowledge the use of AI to assist in language refinement.

## Data Availability

Data sharing is not applicable to this article as no new data were created or analyzed in this study.
